# A cross-sectional study on the quality and reliability of short videos related to precancerous cervical lesions on major platforms

**DOI:** 10.3389/fmed.2026.1917426

**Published:** 2026-09-14

**Authors:** Yuping Shan, Jitong An, Hanxue Zong, Yunyan Ma, Guangjie Yin

**Affiliations:** Department of Gynecology, The Affiliated Hospital of Qingdao University, Qingdao, China

**Keywords:** platform governance, precancerous cervical lesions, public health education, short-video platforms, video quality assessment

## Abstract

**Objective:**

To evaluate the quality, reliability, and educational value of videos related to precancerous cervical lesions across four major short-video platforms in China (TikTok, Bilibili, Kwai, and Xiaohongshu), using multiple validated evaluation tools.

**Methods:**

A total of 1842 short videos were collected, assessed using GQS, JAMA, modified DISCERN, PEMAT, and HONcode tools. The videos were analyzed for quality, reliability, educational value, and engagement metrics (likes, comments, collections, shares).

**Results:**

Videos on TikTok consistently achieved the highest scores for quality, reliability, and transparency across all scoring systems. In contrast, Bilibili, Kwai, and Xiaohongshu exhibited variable performance. Videos created by medical practitioners, particularly gynecologists, scored higher compared to those uploaded by non-medical practitioners. Content focusing on basic disease knowledge had higher educational value than prevention or post-treatment videos. Audience engagement did not always correlate with higher quality.

**Conclusion:**

Short-video platforms may serve as valuable channels for health information dissemination. Among the sampled videos, TikTok demonstrated higher quality scores than the other evaluated platforms. Future efforts may focus on improving health information quality assurance, encouraging healthcare professional participation in content creation, and optimizing video formats for actionable health guidance.

## 1 Introduction

Cervical cancer is a significant global public health challenge and ranks as the third most common malignant tumor among women worldwide in both incidence and mortality (1). Although the incidence of cervical cancer has declined in many regions over the past three decades, it remains a major threat to women's health worldwide, with approximately 604,000 new cases and 280,000 deaths estimated globally in 2024 (2). Over 90% of cervical cancer cases are caused by persistent human papillomavirus (HPV) infection, predominantly types 16 and 18 (3). The majority of HPV infections are asymptomatic and resolve spontaneously within 12–24 months after infection. However, a small subset of these infections persist and progress to precancerous cervical lesions, which can eventually develop into cancer (4). Precancerous cervical lesions, also known as cervical intraepithelial neoplasia (CIN), are classified into three grades: CIN 1, CIN 2, and CIN 3 (5). CIN 1 corresponds to low-grade squamous intraepithelial lesions (LSIL) and spontaneously regresses in approximately 80%–90% of cases (6). During this stage, the virus actively replicates, causing mild changes in the cervical epithelial cells (7). CIN 2 and CIN 3 correspond to high-grade squamous intraepithelial lesions (HSIL) (8). Although viral replication is relatively low during these stages, the virus persists due to immune evasion, a dysregulated vaginal microenvironment, and other cofactors (9). Notably, lifestyle behaviors significantly influence the development of precancerous cervical lesions. Factors such as multiple sexual partners, smoking, and contraceptive methods may increase the risk of precancerous cervical lesions by affecting the persistence and progression of HPV infection (10). Although the incidence of precancerous cervical lesions has increased dramatically in recent years, these lesions have a high potential for cure, which can help reduce both the incidence and mortality rates of cervical cancer (11). Public awareness of precancerous cervical lesions has significantly improved in recent years. However, further efforts are needed to enhance understanding of relevant detection methods and risk factors to improve early screening, prevention, and treatment outcomes.

As short video platforms have evolved, information spreads rapidly through social networks. More and more people are turning to social media for health information and knowledge because it effectively removes the physical barriers that have traditionally limited access to healthcare support and resources (12). Currently, TikTok is the leading short-form social media application worldwide, with 780 million daily active users spending an average of 52 min per day watching more than 200 videos (13). Among young people in China, Bilibili is a highly popular short video social media app, boasting 86% of users under 35 and 94 million daily active users (14). Kwai is a major short video platform with a significant user base, boasting around 714 million monthly active users and more than 408 million daily active users worldwide (15). Xiaohongshu, a free social networking platform, has attracted millions of users in China over the past few years and has significantly influenced the lifestyles of young people through user-generated content and authentic sharing (13). There is an increasing amount of content about precancerous cervical lesions on short video platforms, ranging from detailed medical information to personal patient stories. Research has previously demonstrated that the distribution of health information can modify health behaviors, leading to substantial improvements in early disease diagnosis and prevention (12). On the other hand, nonprofessionals and patients might utilize social media for communication and information sharing, which could cause misinformation to spread (16). Research has found that many videos related to medical science do not have a strong scientific foundation, with numerous creators spreading incorrect information to increase traffic and viewer interaction (17). Thus, it is essential to thoroughly evaluate videos on these platforms regarding precancerous cervical lesions to carefully assess their quality and trustworthiness. This is a crucial step in enhancing the effectiveness of public health education and a necessary measure to ensure that the public has access to accurate and reliable health information.

At present, there has been no systematic evaluation of the quality of video content about precancerous cervical lesions on most short video platforms in China. Therefore, this study aimed to evaluate the quality and reliability of videos related to precancerous cervical lesions on several widely used short-video sharing platforms (TikTok, Bilibili, Kwai, Xiaohongshu) and to provide evidence-based recommendations for platform governance and public health communication in China and similar digital environments.

## 2 Materials and methods

### 2.1 Search strategy

Short-video data collection was completed between December 1 and 2, 2025, from the platforms TikTok (https://www.douyin.com), Bilibili (https://www.bilibili.com/), Kwai (https://www.douyin.com), and Xiaohongshu (https://www.xiaohongshu.com). To minimize the bias introduced by personal recommendation algorithms, we created new accounts on each of the platforms mentioned above. Searches were conducted in incognito mode (logged out), with cleared cache and cookies, and with location services disabled. We used the following keywords for our search in above 4 short-video sharing platforms: “宫颈癌前病变” (precancerous cervical lesions), “宫颈上皮内病变” (cervical intraepithelial lesions), “宫颈上皮内瘤变” (cervical intraepithelial neoplasias), “HSIL”, “LSIL”, “CIN1”、“CIN2”, and “CIN3”. The search results from each platform were sorted according to their default ranking algorithms, without applying any preset filters. The top 100 videos returned under the default ranking settings for each search term were then selected. These videos represented high-ranking videos retrieved by the platform search algorithms rather than the entire available content on the platforms. Videos were manually collected from the platforms, and no API or automated web-scraping tools were used. Before quality assessment, visible identifiers that could reveal uploader identity or engagement metrics (including likes, comments, and platform-specific overlays) were manually removed using video editing software. The original video content was not altered.

For the collected short videos, we screened them according to the following inclusion and exclusion criteria. The inclusion criteria were: (1) videos related to precancerous cervical lesions, and (2) videos in Chinese. Exclusion criteria were: (1) duplicate content, (2) advertisements, and (3) videos containing irrelevant content. Videos primarily intended for commercial promotion, including advertisements for hospitals, clinics, medical products, or treatment services, were excluded. Educational videos uploaded by healthcare professionals or medical institutions were included if they provided health-related information without explicit promotional purposes.

### 2.2 Data collection

The included videos were independently reviewed by two gynecologists, who recorded the relevant information in Microsoft Excel (Microsoft Corporation). Reviewers were first informed about the study and then provided their consent after receiving and understanding the study information. For the included videos, the recorded and analyzed characteristics are platforms, publication date, number of uploaders’ fans, days since upload, video duration, number of likes, number of comments, number of collections, number of shares, uploader types, video content, and video presentation formats.

### 2.3 Variable classification

Categorical variables were classified according to predefined criteria established before data analysis. Uploader type was categorized into five groups: gynecologists, non-gynecologists, traditional Chinese medicine practitioners, science communicators, and patients or family members. Video content was classified into five categories: basic knowledge about diseases, prevention, treatment, posttreatment caveats and reexamination, and others. Video presentation form was classified into six categories: expert monologues, dialogues, video blogs of patients, visual pictures and literature, surgical operation demonstrations, and others. Detailed category definitions are presented in Supplementary Table 1.

Continuous variables, including fans, days since upload, video duration, number of likes, number of comments, number of collections, and number of shares, were recorded as numerical variables. Publication year was derived from the publication date. Engagement rate was calculated as the sum of likes, comments, collections, and shares divided by the number of fans of the video uploader. This follower-based metric was used to normalize audience interactions according to the potential audience size of each uploader and has been commonly applied in social media research. Although alternative metrics based on views or impressions are also available, follower-based engagement provides a comparable measure across accounts with different audience sizes.

### 2.4 Quality and reliability assessment

Video quality and reliability were evaluated using the Global Quality Score (GQS), the JAMA Benchmarking Criteria, the modified DISCERN (mDISCERN) tool, the Patient Education Materials Assessment Tool (PEMAT), and HONcode certification. GQS is a widely used five-point scale that provides an overall subjective judgment of the educational quality and usefulness of online health-related videos (18). The JAMA Benchmark Criteria assess the credibility of online medical information based on four essential components: authorship, attribution, disclosure, and currency (19). The mDISCERN instrument, adapted from the original DISCERN questionnaire, evaluates the reliability of health information by examining whether sources are cited, whether information is balanced and unbiased, and whether uncertainties are acknowledged (12). PEMAT objectively measures the understandability and actionability of patient education materials, determining whether the content can be easily understood by patients and whether it provides clear steps for taking action (20). The Health On the Net (HON) code, developed by the Health On the Net Foundation, was originally established to evaluate the reliability and ethical standards of online health information websites based on eight principles, including authority, complementarity, privacy, attribution, justifiability, transparency, financial disclosure, and advertising policy (13). In this study, the HONcode criteria were adapted to assess the credibility-related characteristics of health information videos on short-video platforms. Considering the differences between websites and video-based social media content, only criteria that could be objectively evaluated from publicly available video information were applied. Specifically, the evaluated domains included authority (whether the creator's professional identity or expertise was disclosed), complementarity (whether the video indicated that online information should not replace professional medical consultation), attribution and transparency (whether information sources and authorship were provided or identifiable), and disclosure-related information when available. Criteria that were not applicable to individual short videos, such as website ownership, editorial responsibility of the entire platform, and site-level privacy policies, were excluded. Together, these tools provide a comprehensive and multidimensional assessment of both the quality and the reliability of the included video materials. Two evaluators independently assessed all included videos. Before consensus discussion, inter-rater agreement between the two reviewers was assessed using weighted Cohen's *κ* coefficients for all quality assessment instruments. Any discrepancies were subsequently resolved through discussion with a third senior gynecologist until consensus was achieved.

### 2.5 Statistical analysis

Categorical variables are presented as frequencies and percentages, with chi-square tests or Fisher's exact tests performed as appropriate. Continuous variables with a normal distribution are expressed as the mean ± standard deviation (SD) and analyzed using Student's *t*-test. For comparisons between two independent groups, Student's t-test or the Mann–Whitney U test was used, as appropriate. For comparisons among three or more independent groups, one-way analysis of variance (ANOVA) or the Kruskal–Wallis test was used, as appropriate. For significant Kruskal–Wallis tests, Dunn's *post hoc* test with Bonferroni adjustment was performed for pairwise comparisons. Double-tailed *p*-value < 0.05 is considered statistically significant. Two reviewers independently assessed all included videos using the predefined evaluation instruments. Inter-rater agreement between reviewers was evaluated using Cohen's *κ* statistics. The *κ* values were interpreted according to the Landis and Koch criteria (21). The outcomes included GQS, JAMA, modified DISCERN, PEMAT understandability, PEMAT actionability, and HONcode scores. The explanatory variables and potential confounders included platform, publication year, uploader type, video content, video presentation format, and video duration. For GQS, JAMA, modified DISCERN, and HONcode scores, which are ordinal outcomes, multivariable ordinal logistic regression analyses were performed to identify factors independently associated with higher assessment scores after adjustment for potential confounding variables. For PEMAT understandability and actionability scores, multivariable linear regression analyses were performed. All explanatory variables and potential confounders were simultaneously included in the respective multivariable models. The Spearman test was applied to evaluate the correlation between various scores and video features. All statistical analyses were performed with SPSS software (version 29.0; IBM Corp., USA).

## 3 Results

### 3.1 General characteristics of included videos

After removing 42.4% duplicate, irrelevant, and advertisement videos according to the predefined exclusion criteria, a total of 1,842 short videos related to precancerous cervical lesions were included in the final analysis. Among the included videos, 565 were obtained from TikTok, 280 from Bilibili, 409 from Kwai, and 588 from Xiaohongshu (Figure 1).

**Figure 1:**
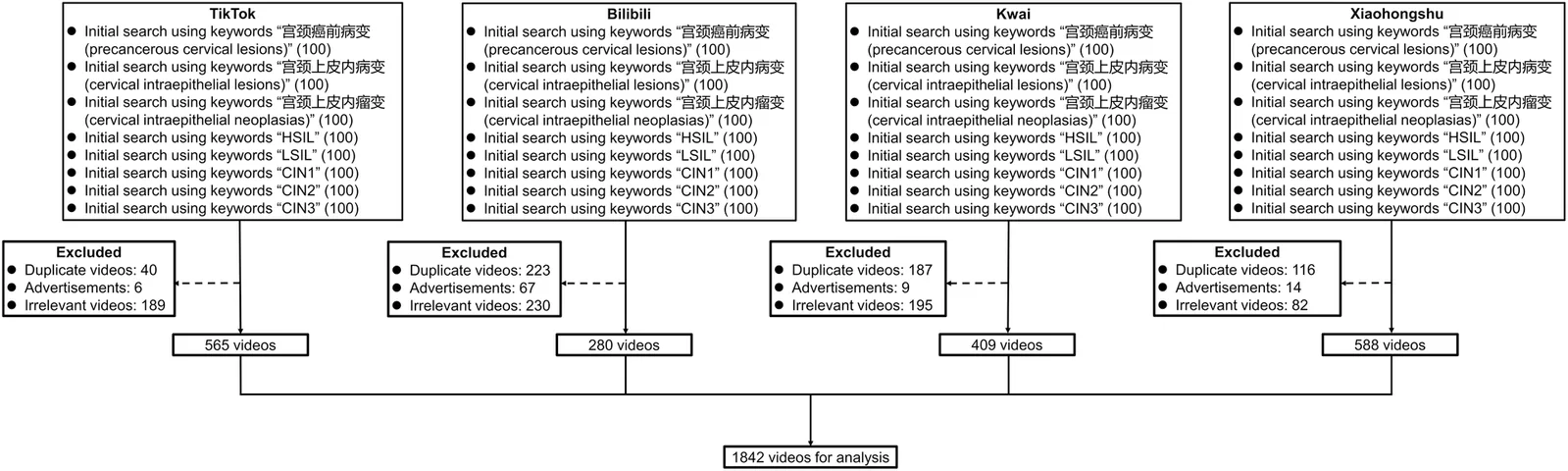
Flowchart of video selection process.

Significant differences were observed among platforms with respect to uploader characteristics, video duration, and audience engagement metrics (Supplementary Table 2). Overall, the median number of uploader followers was 2,700 (IQR, 2013-213995). TikTok demonstrated the highest median follower count, whereas Bilibili exhibited the lowest. The median video duration was 61 s (IQR, 42−89), with videos on Bilibili being significantly longer than those on the other platforms.

Regarding audience engagement, TikTok consistently showed the highest median numbers of likes, comments, collections, and shares, followed by Kwai, while Bilibili displayed the lowest engagement levels across all indicators. These findings indicate marked platform-specific differences in dissemination patterns and user interaction behaviors.

### 3.2 Uploader identity, content categories, and presentation formats

As shown in Supplementary Table 3, the majority of videos were uploaded by medical practitioners (95.8%), with gynecologists accounting for 91.5% of all uploaders. Non-medical practitioners represented only a small proportion (4.2%), including science communicators and patients or their family members. The distribution of uploader identities differed significantly across platforms, with all videos on Kwai uploaded by medical professionals.

In terms of content categories, treatment-related videos constituted the largest proportion (53.4%), followed by videos providing basic disease knowledge (34.4%). Videos focusing on prevention (2.9%) and post-treatment precautions or follow-up (2.8%) were relatively uncommon. Content distribution varied significantly among platforms, with TikTok and Kwai featuring a higher proportion of basic disease knowledge videos, whereas Bilibili predominantly focused on treatment-related content.

Regarding presentation formats, expert monologues were the most prevalent (67.7%), followed by dialogues (25.0%). Patient video blogs, visual illustrations, and surgical operation demonstrations together accounted for a relatively small proportion of the total videos. Significant differences in presentation formats were observed across platforms.

### 3.3 Quality, reliability, and educational value of videos

The agreement between the two reviewers was substantial across all assessment instruments. The Cohen's *κ* values ranged from 0.68 to 0.81, indicating good reproducibility of the quality assessment results (Table 1). Videos published on TikTok achieved the highest median scores in the Global Quality Score (GQS), JAMA Benchmark Criteria, modified DISCERN, and HONcode, indicating superior overall quality, reliability, and transparency compared with other platforms. Kwai demonstrated comparable performance to TikTok in terms of PEMAT understandability and actionability scores, suggesting strong patient-oriented educational characteristics. However, its performance in transparency-related indicators, including JAMA and HONcode scores, was inferior to that observed on TikTok. In contrast, videos on Bilibili and Xiaohongshu consistently exhibited lower scores across multiple quality and reliability metrics.

**Table 1:** Inter-rater agreement between two reviewers for video quality assessment.

Assessment tool	Cohen's kappa (*κ*)	95% CI	Agreement
GQS	0.74	0.72–0.89	Substantial
JAMA score	0.81	0.59–0.84	Almost perfect
Modified DISCERN score	0.77	0.74–0.92	Substantial
PEMAT Understandability score	0.71	0.68–0.73	Substantial
PEMAT Actionability score	0.68	0.45–0.71	Substantial
HONCODE score	0.79	0.76–0.80	Almost perfect

Agreement was interpreted according to the Landis and Koch criteria: κ < 0.20, poor agreement; 0.21–0.40, fair agreement; 0.41–0.60, moderate agreement; 0.61–0.80, substantial agreement; and 0.81–1.00, almost perfect agreement.

Supplementary Figures 1–6 illustrate the distributions of GQS, JAMA, modified DISCERN, PEMAT understandability, PEMAT actionability, and HONcode scores according to uploader characteristics, video content, and video presentation format. To further identify factors independently associated with higher assessment scores, multivariable regression analyses were performed. After mutual adjustment for platform, publication year, uploader type, video content, video presentation format, and video duration, platform, publication year, and uploader type were independently associated with the six assessment outcomes (Supplementary Tables 4–9). Compared with TikTok, videos on Bilibili, Kwai, and Xiaohongshu generally received lower assessment scores, although the significance of individual platform comparisons varied across assessment domains. Assessment scores generally increased with publication year across all six outcomes, indicating that more recently published videos tended to have higher scores. Uploader type was also independently associated with the assessment outcomes. Compared with videos uploaded by gynecologists, videos uploaded by traditional Chinese medicine practitioners, science communicators, and patients and family members generally received lower scores, whereas differences between gynecologists and non-gynecologists varied across assessment domains. Video content was significantly associated with JAMA, PEMAT understandability, PEMAT actionability, and HONcode scores. Compared with videos focusing on basic disease knowledge, videos addressing prevention, treatment, post-treatment precautions and follow-up examinations, or other topics generally showed lower scores in the assessment domains for which significant associations were observed. Video presentation format was significantly associated with GQS, JAMA, modified DISCERN, PEMAT understandability, PEMAT actionability, and HONcode scores, with several formats, particularly patient video blogs and other formats, showing lower scores than expert monologues.

### 3.4 Association between uploader characteristics, content type, and audience engagement

Audience engagement metrics stratified by uploader characteristics, content categories, and presentation formats are summarized in Supplementary Table 10. No statistically significant differences were observed in likes, comments, collections, or shares between videos uploaded by medical practitioners and non-medical practitioners, although the number of videos uploaded by non-medical practitioners was relatively small. In contrast, substantial differences in engagement were observed among uploader subtypes. Videos uploaded by patients and family members received significantly higher numbers of likes, comments, collections, and shares compared with those uploaded by other uploader groups. Engagement metrics also differed significantly across video content categories, with prevention-focused videos consistently showing the lowest engagement levels. Regarding presentation formats, patient video blogs and surgical operation demonstrations generated markedly higher engagement compared with expert monologues, dialogues, and visual illustration formats.

### 3.5 Relationship between audience engagement and informational quality

Figure 2 illustrates the correlation patterns, displaying correlation coefficients and their corresponding significance levels. Spearman correlation analysis showed that the number of fans was significantly associated with all six quality and reliability measures, including GQS, JAMA, modified DISCERN, PEMAT understandability, PEMAT actionability, and HONcode scores. Similarly, likes, comments, collections, and shares were significantly associated with all six quality and reliability measures. Days since publication was significantly associated with GQS, modified DISCERN, PEMAT actionability, and HONcode scores, whereas video duration was significantly associated only with GQS.

**Figure 2:**
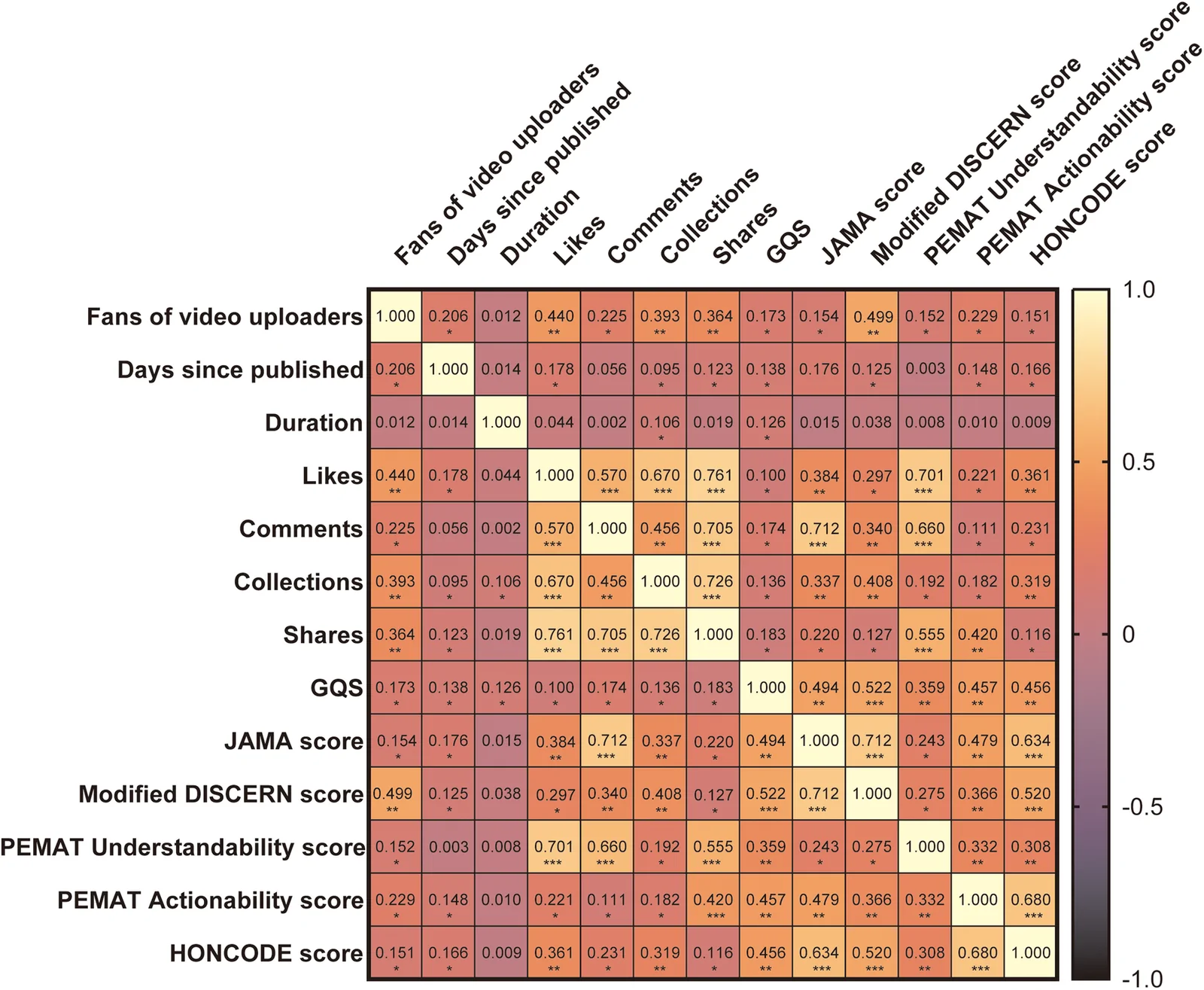
Correlation heatmap of video characteristics, engagement metrics, and quality assessment scores. Statistical significance is indicated by asterisks: *p-value < 0.05, ** *p*-value < 0.01, *** *p*-value < 0.001.

## 4 Discussion

This research offers an in-depth assessment of the quality, reliability, and educational value of short videos concerning precancerous cervical lesions across four prominent short-video platforms in China. Utilizing a range of validated evaluation instruments, such as GQS, JAMA, modified DISCERN, PEMAT understandability, PEMAT actionability, and HONcode, our analysis uncovers significant variability in video quality across different platforms, uploader profiles, content categories, and presentation styles. Similar concerns regarding the quality and reliability of online gynecological health information have also been reported in previous studies, highlighting the challenges of ensuring accurate and accessible medical information dissemination through digital platforms (22). These findings underscore both the potential benefits and the inherent limitations of short-video platforms as sources of health information for the prevention and early intervention of cervical cancer.

A principal finding of this study is the identification of substantial platform-level disparities across all assessed scoring systems. Specifically, videos disseminated on TikTok consistently attained superior scores in overall quality, reliability, transparency, and ethical compliance. In contrast, Bilibili, Kwai, and Xiaohongshu exhibited variable and frequently lower performance across different evaluative metrics. These results align with prior research, which has similarly documented significant differences in informational quality across various platforms (12).

Several factors may account for the observed differences specific to each platform. Primarily, the governance mechanisms employed by these platforms, such as content review policies, creator credential verification processes, and strategies for moderating medical content, differ significantly across platforms. Differences in platform policies, creator composition, and content distribution mechanisms may contribute to the observed platform-level differences (23). Conversely, platforms that employ algorithms with a stronger focus on entertainment may prioritize engagement metrics over informational accuracy, potentially compromising the overall quality of health-related content.

Secondly, variations in user demographics and content consumption behaviors across platforms can indirectly influence the characteristics of uploaded content. Platforms like Kwai and Xiaohongshu, which predominantly attract younger audiences and focus on lifestyle-oriented content, may encourage the creation of simplified or experience-based narratives rather than detailed medical explanations (20). This trend has been documented in research examining short videos related to sexually transmitted diseases and oncology, where increased user engagement did not always correlate with higher informational quality (14, 24).

Across various platforms, the understandability scores of the PEMAT consistently surpassed the actionability scores, suggesting that although most videos demonstrated acceptable understandability., they frequently lacked clear, actionable guidance for viewers. This trend is consistent with previous research on short-video health communication, which indicates that content creators often prioritize explaining disease concepts over providing concrete guidance on health behaviors (25).

For precancerous cervical lesions, inadequate actionability can have significant public health consequences. The effective prevention and management of these lesions are highly dependent on timely screening, adherence to follow-up schedules, lifestyle modifications, and appropriate treatment decisions. Videos that do not provide explicit guidance on when to seek medical care, how to interpret screening results, or how to adhere to follow-up recommendations may diminish their practical utility, despite the accuracy and clarity of the information presented. This disparity between understandability and actionability highlights the necessity for targeted content optimization. Encouraging content creators, particularly medical professionals, to integrate practical recommendations, decision cues, and behavioral prompts could substantially enhance the educational impact of short-video health content.

The differences in video quality according to uploader characteristics may be related to differences in professional expertise, access to evidence-based medical information, and familiarity with clinical guidelines. Medical professionals may be better positioned to provide accurate and appropriately contextualized health information, consistent with previous research showing that professional medical backgrounds are associated with improved accuracy and credibility of online health information (26). In contrast, patient- and family-generated content may place greater emphasis on personal experiences and emotional communication. This distinction between informational quality and audience engagement is consistent with previous research on short-video health communication, in which personal narratives and emotional storytelling may attract greater interaction while providing less comprehensive or balanced medical information. Nevertheless, patient-generated videos can offer emotional support and foster peer connections. Therefore, platform governance strategies should prioritize collaboration between medical professionals and patient content creators rather than simply restricting non-professional content. Such collaboration could help ensure that experiential narratives are presented alongside accurate medical context, thereby balancing credibility with relatability.

The observed differences across video content categories may reflect the varying challenges involved in presenting different types of health information in a concise and accessible format. The substantial underrepresentation of prevention- and follow-up-related content identified in this study highlights an important gap in current online health communication. Preventive information, such as HPV vaccination, cervical cancer screening, risk factor modification, and follow-up recommendations, often requires the integration of evidence-based guidelines into concise and accessible formats, which may present greater challenges for content creators (26). To address this gap, authoritative medical institutions, including national cancer centers and specialized gynecologic oncology departments, could actively participate in the development and dissemination of evidence-based short videos focusing on cervical cancer prevention and early detection. Such institution-led initiatives may help improve the balance between treatment-oriented and prevention-oriented content while providing the public with more actionable health information. Furthermore, considering that TikTok demonstrated relatively higher quality and reliability scores among the evaluated platforms, collaboration between professional medical organizations and mainstream short-video platforms may represent a potential strategy to enhance the accessibility and visibility of high-quality cervical cancer prevention information.

Regarding presentation format, expert monologues remained independently associated with higher quality scores after adjustment. This may be explained by the structured nature of expert-led explanations, which facilitates accurate information delivery and improves clarity. However, formats that promote audience interaction, such as patient video blogs, may generate greater engagement despite potentially lower informational quality. In contrast, patient video blogs and surgical demonstrations were linked to increased engagement. This disparity underscores an intrinsic structural tension within short-video health communication: the formats that optimize engagement do not invariably provide the most reliable information. To rectify this imbalance, it may be beneficial to develop content formats that integrate professional medical expertise with engaging storytelling approaches, thereby improving both the reliability and accessibility of short-video health information.

Our correlation analysis has revealed a significant association between audience engagement metrics and video quality or reliability scores. This finding is consistent with previous research on short-video health content, which indicates that higher audience engagement is generally correlated with higher quality scores (27). Additionally, our findings suggest that engagement-based algorithms may partially enhance the visibility of high-quality videos, as some engagement indicators were positively associated with specific quality dimensions. However, audience engagement should not be considered a direct proxy for informational quality, because highly interactive content may not always provide accurate or comprehensive health information. Therefore, recommendation systems should incorporate more precise quality assessment indicators beyond engagement metrics. Such efforts may require collaboration among social media platforms, healthcare professionals, and user communities, including platform-based quality evaluation, professional review, and community moderation approaches such as reporting misleading content and providing fact-checking feedback.

In summary, our findings underscore the dual function of short-video platforms as both potent instruments for health education and potential conduits of misinformation. For precancerous cervical lesions, a condition characterized by high preventability and treatability, ensuring the availability of accurate, reliable, and actionable information is of paramount importance. It is imperative for platform operators to implement more rigorous verification mechanisms for medical content, enhance the visibility of high-quality videos through algorithmic prioritization, and offer incentives for professional creators to incorporate actionable guidance. From a public health standpoint, integrating short-video platforms into cervical cancer prevention strategies could enhance awareness and increase screening uptake, contingent upon the rigorous monitoring and optimization of content quality.

### 4.1 Limitation

This study is subject to several limitations. Firstly, the sampling strategy may introduce selection bias. Since videos were collected based on the default ranking algorithms of each platform, the included videos mainly represented high-ranking videos returned by platform search systems and may not fully represent all available content on these platforms. Secondly, the included videos were limited to Chinese-language health information videos from Chinese short-video platforms. Therefore, the findings may not be directly generalizable to other social media platforms, such as YouTube, Instagram, Facebook, or Western versions of TikTok, where user demographics, content regulation, recommendation algorithms, and health communication environments may differ substantially. Thirdly, the majority of included videos were uploaded by medical practitioners, which may have limited the representativeness of our sample and potentially resulted in an overestimation of the overall quality and reliability of cervical lesion-related health information. Future studies should include a broader range of content creators, including patient-generated and non-professional content, to better capture the diversity of health information available on short-video platforms.

### 4.2 Future research

Future research should investigate longitudinal changes in health-related short-video content quality as platform algorithms, user behaviors, and regulatory environments continue to evolve. In addition, multicenter and cross-cultural investigations involving different countries and healthcare systems are needed to determine whether these findings can be generalized beyond Chinese-language platforms. Future studies may further evaluate interventions aimed at improving online health communication, including collaborations between medical institutions and short-video platforms, professional content certification initiatives, and community-based fact-checking mechanisms. Furthermore, artificial intelligence-based approaches for large-scale monitoring, misinformation detection, and automated quality assessment may provide promising tools for supporting the evaluation and management of digital health information.

## 5 Conclusion

This study offers a comprehensive assessment of the quality, reliability, and educational value of videos pertaining to precancerous cervical lesions across four prominent short-video platforms in China. The findings indicate significant platform-specific disparities in video quality, with TikTok generally receiving the highest scores. In contrast, Bilibili, Kwai, and Xiaohongshu displayed varying degrees of quality and reliability. Videos produced by medical professionals, particularly gynecologists, were of superior quality compared to those created by non-medical practitioners. Furthermore, although content focused on fundamental disease knowledge received higher scores, prevention-related videos were substantially underrepresented, highlighting the need for greater involvement of professional medical institutions in developing accessible and actionable cervical cancer prevention content. Importantly, likes, comments, collections, and shares were all significantly associated with the six quality and reliability measures, including GQS, JAMA, modified DISCERN, PEMAT understandability, PEMAT actionability, and HONcode. These findings suggest that audience engagement may be related to the informational quality of short videos and should be considered when developing platform recommendation and quality evaluation strategies. These findings highlight the critical need to enhance platform governance, content moderation, and the engagement of healthcare professionals in content creation to ensure the dissemination of precise and actionable health information. As short-video platforms assume an increasingly pivotal role in health communication, optimizing these platforms for accurate health education, particularly in the context of cervical cancer prevention and early detection, could significantly bolster public health initiatives.

## Data Availability

The original contributions presented in the study are included in the article/Supplementary Material, further inquiries can be directed to the corresponding author.

## References

[1] Ogasawara A Hasegawa K. Recent advances in immunotherapy for cervical cancer. Int J Clin Oncol. (2025) 30(3):434–48. 10.1007/s10147-025-02699-0 39888529 PMC11842527

[2] World Health Organization. Cervical Cancer. Geneva: World Health Organization (2026).

[3] Voelker RA. Cervical cancer screening. Jama. (2023) 330(20):2030. 10.1001/jama.2023.21987 37889510

[4] Hou Y Ni S Liu X Liu X Wang N Xu F. HPV Integration profiling using nanopore sequencing and association with cervical precancerous lesion. Front Microbiol. (2025) 16:1522550. 10.3389/fmicb.2025.1522550 40109972 PMC11920162

[5] Ye J Zheng L He Y Qi X. Human papillomavirus associated cervical lesion: pathogenesis and therapeutic interventions. MedComm. (2023) 4(5):e368. 10.1002/mco2.368 37719443 PMC10501338

[6] Loopik DL Bentley HA Eijgenraam MN IntHout J Bekkers RLM Bentley JR. The natural history of cervical intraepithelial neoplasia grades 1, 2, and 3: a systematic review and meta-analysis. J Low Genit Tract Dis. (2021) 25(3):221–31. 10.1097/LGT.0000000000000604 34176914

[7] Piyathilake CJ Badiga S Simons JL Bell WC Jolly PE. HPV E1 qPCR, a low-cost alternative assay to roche diagnostic linear array is effective in identifying women at risk for developing cervical cancer. Int J Womens Health. (2022) 14:257–66. 10.2147/IJWH.S347546 35221728 PMC8865867

[8] Tango CN Seo SS Kwon M Lee DO Chang HK Kim MK. Taxonomic and functional differences in cervical microbiome associated with cervical cancer development. Sci Rep. (2020) 10(1):9720. 10.1038/s41598-020-66607-4 32546712 PMC7297964

[9] Zeng Y Jiang T Zheng Y Yang J Wei H Yi C. Risk factors predicting residual lesion in subsequent hysterectomy following cold knife conization (CKC) for high-grade squamous intraepithelial lesion (HSIL). BMC Women’s Health. (2022) 22(1):358. 10.1186/s12905-022-01939-z PMC9426006 36042513

[10] Huang Y Wu X Lin Y Li W Liu J Song B. Multiple sexual partners and vaginal microecological disorder are associated with HPV infection and cervical carcinoma development. Oncol Lett. (2020) 20(2):1915–21. 10.3892/ol.2020.11738 32724435 PMC7377087

[11] Samiee Rad F Ghaebi M Zarabadipour S Bajelan A Pashazade F Kalhor M. Comparison of diagnostic methods in detection of squamous cell abnormalities in Iranian women with abnormal pap’s smear test and associated demographic and issues. Iran J Pathol. (2020) 15(2):106–16. 10.30699/ijp.2020.114626.2248 32215026 PMC7081767

[12] Zhang J Yuan J Zhang D Yang Y Wang C Dou Z. Short video platforms as sources of health information about cervical cancer: a content and quality analysis. PLoS One. (2024) 19(3):e0300180. 10.1371/journal.pone.0300180 38457419 PMC10923403

[13] Qi X Lu Q Li B Liu S Zhang L Xiang R. Videos in short video sharing platforms as a source of information on bipolar disorder: a cross-sectional content analysis study. Front Public Health. (2025) 13:1627885. 10.3389/fpubh.2025.1627885 41229485 PMC12602428

[14] Peng J Tu M Li S Ding X Zhai Q Wu H. Assessment of the reliability and quality of pancreatic cancer related short videos on mainstream platforms: cross-sectional study. BMC Cancer. (2025) 25(1):1428. 10.1186/s12885-025-14825-2 40993566 PMC12461966

[15] Su L Yao J Ai X Ren Y Xu R Wu X. Short video platforms as sources of health information about HPV vaccine: a content and quality analysis. Digit Health. (2025) 11:20552076251379340. 10.1177/20552076251379340 40964602 PMC12437182

[16] O'Connor S Odewusi T Smith PM Booth RG. Digital professionalism on social media: the opinions of undergraduate nursing students. Nurse Educ Today. (2022) 111:105322. 10.1016/j.nedt.2022.105322 35263709

[17] Zhang YX Yin HT Liu YX Fu X Liu J. Videos in short-video sharing platforms as sources of information on osteoarthritis: cross-sectional content analysis study. Front Digit Health. (2025) 7:1622503. 10.3389/fdgth.2025.1622503 40978700 PMC12446246

[18] Liu ZY Chen YW Lin Y Ai MM Lian DL Zhang YH. YouTube/ bilibili/ TikTok videos as sources of medical information on laryngeal carcinoma: cross-sectional content analysis study. BMC Public Health. (2024) 24(1):1594. 10.1186/s12889-024-19077-6 38877432 PMC11177428

[19] Kwan JY Stocco F Scott DJA Bailey MA Coughlin PA. Assessment of internet-based information on statin therapy. Eur J Cardiovasc Nurs. (2024) 23(2):115–21. 10.1093/eurjcn/zvad061 37367216

[20] Guan J-L Xia S-H Zhao K Feng L-N Han Y-Y Li J-Y. Videos in short-video sharing platforms as sources of information on colorectal polyps: cross-sectional content analysis study. J Med Internet Res. (2024) 26:e51655. 10.2196/51655 39470708 PMC11558218

[21] Landis JR Koch GG. The measurement of observer agreement for categorical data. Biometrics. (1977) 33(1):159–74. 10.2307/2529310 843571

[22] Libretti A Vitale SG Saponara S Corsini C Aquino CI Savasta F. Hysteroscopy in the new media: quality and reliability analysis of hysteroscopy procedures on YouTube™. Arch Gynecol Obstet. (2023) 308(5):1515–24. 10.1007/s00404-023-07172-9 37568070 PMC10520162

[23] Zhu W He B Wang X Du Y Young K Jiang S. Information quality of videos related to esophageal cancer on tiktok, kwai, and bilibili: a cross-sectional study. BMC Public Health. (2025) 25(1):2245. 10.1186/s12889-025-23475-9 40604838 PMC12220660

[24] Zhao K Yang J Liu J. The quality and content of gonorrhea-related short videos on TikTok: a cross-sectional study. Digit Health. (2025) 11:20552076251391170. 10.1177/20552076251391170 41181551 PMC12575989

[25] Li Z Zhong Z Deng X Liao F Hu Y Liu X. Assessment of information quality and reliability on ankle sprains in short videos from douyin and bilibili. Sci Rep. (2025) 15(1):22654. 10.1038/s41598-025-07656-5 40596304 PMC12218369

[26] Xie X Li Y Chen Z Zhou P Jin X. Quality and accuracy of cardiopulmonary resuscitation teaching in short videos: an analysis across three major short video platforms. BMC Med Educ. (2025) 25(1):631. 10.1186/s12909-025-06776-w 40301843 PMC12042457

[27] Zhou Q Shen J Cao D Yu W Chen J. Quality and accuracy of scoliosis-related short videos on TikTok reviewed in China in August 2025. Sci Rep. (2025) 15(1):44007. 10.1038/s41598-025-27684-5 41408068 PMC12711883

