# Supplementary material for: A cross-sectional study on the quality and reliability of short videos related to precancerous cervical lesions on major platforms
Source: Front Med (Lausanne). 2026 Sep 14;13:1917426. doi: 10.3389/fmed.2026.1917426 (PMC13617106; doi:10.3389/fmed.2026.1917426)
Supplement: Supplementary file 1 [file Datasheet1.docx]

**Supplementary Table 1.** Study variables, categories, and definitions.

| Variable | Category | Definition |
| --- | --- | --- |
| Platforms | TikTok | - |
|  | Bilibili | - |
|  | Kwai | - |
|  | Xiaohongshu | - |
| Uploader types | Gynecologists | Western medicine specialists whose primary clinical practice is gynecology |
|  | Non-gynecologists | Western medicine health professionals whose specialty is not gynecology, including general practitioners, oncologists, and other clinicians |
|  | Traditional Chinese medicine practitioners | Physicians formally trained in and practicing traditional Chinese medicine |
|  | Science communicators | Individual creators or representatives of nonprofit organizations or institutions primarily engaged in health education and science popularization |
|  | Patients and family members | Individuals with precancerous cervical lesions or their relatives who share disease-related information or personal experiences |
| Video content | Basic knowledge about diseases | Anatomical, pathological, epidemiological, or fundamental research information related to precancerous cervical lesions |
|  | Prevention | Risk factor control, screening recommendations, and other preventive information |
|  | Treatment | Medical or surgical management, treatment options, procedures, efficacy, and potential adverse effects |
|  | Posttreatment caveats and reexamination | Postoperative or post-treatment care, lifestyle or precautionary advice, and follow-up visits or examinations |
|  | Others | Content that cannot be clearly assigned to the above categories or involves multiple topics without a dominant theme |
| Video presentation form | Expert monologues | A single health professional or science communicator explains the topic in a lecture- or talk-style format |
|  | Dialogues | At least two speakers discuss issues related to precancerous cervical lesions |
|  | Video blogs of patients | Patients or family members share personal experiences, feelings, or treatment journeys |
|  | Visual pictures and literature | Information presented mainly through animations, cartoons, infographics, text slides, or excerpts from guidelines or scientific literature |
|  | Surgical operation demonstrations | Real or simulated surgical procedures related to diagnosis or treatment |
|  | Others | Other formats, such as news clips, entertainment excerpts, or reposted videos without a clear presenter |
| Fans | Continuous | Number of followers of the uploader |
| Publication years | Continuous | Year in which the video was published |
| Days since upload | Continuous | Number of days from publication to the date of data collection |
| Video duration | Continuous | Duration of the video |
| Likes | Continuous | Number of likes |
| Comments | Continuous | Number of comments |
| Collections | Continuous | Number of collections |
| Shares | Continuous | Number of shares |

**Supplementary Table 2.** General characteristics and quality assessment scores of short videos related to precancerous cervical lesions across different platforms.

| Variables | Overall  (N = 1842) | TikTok  (N = 565) | Bilibili  (N = 280) | Kwai  (N = 409) | Xiaohongshu  (N = 588) |
| --- | --- | --- | --- | --- | --- |
| Fans, median (IQR) | 2700 (2013, 213995) | 213986 (53446, 249264) | 1394 (473, 2894) | 12000 (1968, 27000) | 14100 (29000, 544000) |
| Days since published, median (IQR) | 476 (209, 824) | 330 (174, 546) | 633 (312, 1,030) | 631 (434, 1,106) | 452 (113, 997) |
| Duration (seconds), median (IQR) | 61 (42, 89) | 55 (40, 86) | 73 (48, 98) | 58 (41, 85) | 64 (44, 90) |
| Likes, median (IQR) | 111 (9, 475) | 561 (215, 1441) | 17 (6, 56) | 295(145, 550) | 122 (89, 393) |
| Comments, median (IQR) | 11 (1, 72) | 107 (32, 285) | 2 (0, 9) | 24 (9, 59) | 8(3, 10) |
| Collections, median (IQR) | 22 (3, 93) | 99 (29, 301) | 1 (0, 2) | 46 (19, 88) | 7 (2, 29) |
| Shares, median (IQR) | 19 (2, 89) | 81 (18, 283) | 4 (2, 9) | 53 (26, 115) | 5 (1, 21) |
| GQS, median (IQR) | 3 (3, 4) | 4 (3, 5) | 2 (2, 3) | 4 (3, 4) | 3 (2, 4) |
| JAMA score, median (IQR) | 2 (2, 3) | 3 (3, 4) | 1 (1, 2) | 2 (2, 2) | 2 (1, 2) |
| Modified DISCERN score, median (IQR) | 3 (2, 3) | 4 (4, 4) | 2 (1, 3) | 3 (2, 3) | 2 (2, 2) |
| PEMAT Understandability score, median (IQR) | 0.77 (0.64, 0.85) | 0.85 (0.77, 0.92) | 0.31 (0.15, 0.46) | 0.85 (0.77, 0.92) | 0.73 (0.64, 0.82) |
| PEMAT Actionability score, median (IQR) | 0.50 (0.25, 0.75) | 0.75 (0.75, 1.00) | 0.25 (0.25, 0.50) | 0.75 (0.50, 0.75) | 0.25 (0.00, 0.25) |
| HONCODE score, median (IQR) | 3 (2, 5) | 6 (5, 6) | 2 (2, 3) | 4 (3, 4) | 2 (2, 3) |

**Supplementary Table 3.** Uploader characteristics, content categories, and presentation formats of short videos on precancerous cervical lesions.

| Variables | Overall  (N = 1842) | TikTok  (N = 565) | Bilibili  (N = 280) | Kwai  (N = 409) | Xiaohongshu  (N = 588) |
| --- | --- | --- | --- | --- | --- |
| Medical practitioners |  |  |  |  |  |
| Yes | 1764 (95.8%) | 549 (97.2%) | 257 (91.8%) | 409 (100.0%) | 549 (93.4%) |
| No | 78 (4.2%) | 16 (2.8%) | 23 (8.2%) | 0 (0.0%) | 39 (6.6%) |
| Uploader types, number (percentage) |  |  |  |  |  |
| Gynecologists | 1685 (91.5%) | 547 (96.8%) | 222 (79.3%) | 409 (100.0%) | 507 (86.2%) |
| Non-gynecologists | 41 (2.2%) | 5 (0.9%) | 26 (9.3%) | 0 (0.0%) | 10 (1.7%) |
| Traditional Chinese medical experts | 47 (2.6%) | 0 (0.0%) | 14 (5.0%) | 0 (0.0%) | 33 (5.6%) |
| Science communicators | 34 (1.9%) | 0 (0.0%) | 15 (5.4%) | 0 (0.0%) | 19 (3.2%) |
| Patients and family members | 35 (1.9%) | 13 (2.3%) | 3 (1.1%) | 0 (0.0%) | 19 (3.2%) |
| Video content, number (percentage) |  |  |  |  |  |
| Basic knowledge about diseases | 633 (34.4%) | 285 (50.4%) | 38 (13.6%) | 219 (53.6%) | 91 (15.5%) |
| Prevention | 54 (2.9%) | 4 (0.7%) | 23 (8.2%) | 8 (2.0%) | 19 (3.2%) |
| Treatment | 984 (53.4%) | 252 (44.6%) | 210 (75.0%) | 179 (43.8%) | 343 (58.3%) |
| Posttreatment caveats and reexamination | 52 (2.8%) | 21 (3.7%) | 3 (1.1%) | 3 (0.7%) | 25 (4.3%) |
| Others | 119 (6.5%) | 3 (0.5%) | 6 (2.1%) | 0 (0.0%) | 110 (18.7%) |
| Video presentation form, number (percentage) |  |  |  |  |  |
| Expert monologs | 1247 (67.7%) | 369 (65.3%) | 144 (51.4%) | 400 (97.8%) | 334 (56.8%) |
| Dialogues | 461 (25.0%) | 144 (25.5%) | 116 (41.4%) | 8 (2.0%) | 193 (32.8%) |
| Video blogs of patients | 38 (2.1%) | 14 (2.5%) | 4 (1.4%) | 1 (0.2%) | 19 (3.2%) |
| Visual pictures and literature | 32 (1.7%) | 5 (0.9%) | 9 (3.2%) | 0 (0.0%) | 18 (3.1%) |
| Surgical operation demonstration | 49 (2.7%) | 32 (5.7%) | 3 (1.1%) | 0 (0.0%) | 14 (2.4%) |
| Others | 15 (0.8%) | 1 (0.2%) | 4 (1.4%) | 0 (0.0%) | 10 (1.7%) |

**Supplementary Table 4.** Multivariable analyses of factors associated with higher GQS among short videos related to precancerous cervical lesions.

| Variables | Multivariable analyses | |
| --- | --- | --- |
|  | OR (95% CI) | *p*-value |
| Platforms |  | <0.001 |
| TikTok | Reference |  |
| Bilibili | 0.24 (0.06, 0.67) |  |
| Kwai | 0.59 (0.17, 0.93) |  |
| Xiaohongshu | 0.46 (0.33, 0.98) |  |
| Publication years (per year increase) | 1.23 (1.11, 1.86) | 0.015 |
| Uploader types |  | 0.046 |
| Gynecologists | Reference |  |
| Non-gynecologists | 0.87 (0.63, 1.20) |  |
| Traditional Chinese medical experts | 0.35 (0.22, 0.67) |  |
| Science communicators | 0.73 (0.51, 0.98) |  |
| Patients and family members | 0.77 (0.29, 0.99) |  |
| Video duration (per minute increase) | - | - |
| Video content | - | - |
| Basic knowledge about diseases |  |  |
| Prevention |  |  |
| Treatment |  |  |
| Posttreatment caveats and reexamination |  |  |
| Others |  |  |
| Video presentation form |  | <0.001 |
| Expert monologs | Reference |  |
| Dialogues | 0.69 (0.01, 0.98) |  |
| Video blogs of patients | 0.71 (0.23, 0.84) |  |
| Visual pictures and literature | 0.97 (0.89, 1.47) |  |
| Surgical operation demonstration | 0.91 (0.53, 0.99) |  |
| Others | 0.31 (0.18, 0.59) |  |

**Supplementary Table 5.** Multivariable analyses of factors associated with higher JAMA scores among short videos related to precancerous cervical lesions.

| Variables | Multivariable analyses | |
| --- | --- | --- |
|  | OR (95% CI) | *p*-value |
| Platforms |  | 0.002 |
| TikTok | Reference |  |
| Bilibili | 0.73 (0.53, 0.96) |  |
| Kwai | 0.84 (0.76, 1.12) |  |
| Xiaohongshu | 0.74 (0.56, 0.95) |  |
| Publication years (per year increase) | 1.14 (1.05, 1.21) | 0.003 |
| Uploader types |  | 0.030 |
| Gynecologists | Reference |  |
| Non-gynecologists | 0.92 (0.23, 1.36) |  |
| Traditional Chinese medical experts | 0.40 (0.31, 0.67) |  |
| Science communicators | 0.67 (0.47, 0.91) |  |
| Patients and family members | 0.71 (0.54, 0.98) |  |
| Video duration (per minute increase) | 1.04 (0.39, 1.06) | 0.229 |
| Video content |  | 0.041 |
| Basic knowledge about diseases | Reference |  |
| Prevention | 0.74 (0.54, 0.90) |  |
| Treatment | 0.33 (0.21, 0.44) |  |
| Posttreatment caveats and reexamination | 0.37 (0.25, 0.68) |  |
| Others | 0.45 (0.36, 0.61) |  |
| Video presentation form |  | 0.020 |
| Expert monologs | Reference |  |
| Dialogues | 0.77 (0.60, 1.04) |  |
| Video blogs of patients | 0.64 (0.46, 0.94) |  |
| Visual pictures and literature | 0.92 (0.73, 0.98) |  |
| Surgical operation demonstration | 0.81 (0.20, 0.89) |  |
| Others | 0.52 (0.34, 0.81) |  |

**Supplementary Table 6.** Multivariable analyses of factors associated with higher modified DISCERN scores among short videos related to precancerous cervical lesions.

| Variables | Multivariable analyses | |
| --- | --- | --- |
|  | OR (95% CI) | *p*-value |
| Platforms |  | 0.008 |
| TikTok | Reference |  |
| Bilibili | 0.66 (0.49, 0.95) |  |
| Kwai | 0.89 (0.66, 1.18) |  |
| Xiaohongshu | 0.72 (0.53, 0.98) |  |
| Publication years (per year increase) | 1.19 (1.08, 1.31) | <0.001 |
| Uploader types |  | 0.043 |
| Gynecologists | Reference |  |
| Non-gynecologists | 0.88 (0.61, 0.95) |  |
| Traditional Chinese medical experts | 0.36 (0.22, 0.50) |  |
| Science communicators | 0.54 (0.38, 0.91) |  |
| Patients and family members | 0.69 (0.42, 0.88) |  |
| Video duration (per minute increase) | 1.55 (0.93, 3.28) | 0.333 |
| Video content | - | - |
| Basic knowledge about diseases |  |  |
| Prevention |  |  |
| Treatment |  |  |
| Posttreatment caveats and reexamination |  |  |
| Others |  |  |
| Video presentation form |  | 0.011 |
| Expert monologs | Reference |  |
| Dialogues | 0.79 (0.56, 1.04) |  |
| Video blogs of patients | 0.51 (0.32, 0.85) |  |
| Visual pictures and literature | 0.80 (0.60, 1.12) |  |
| Surgical operation demonstration | 0.85 (0.23, 0.96) |  |
| Others | 0.44 (0.29, 0.75) |  |

**Supplementary Table 7.** Multivariable analyses of factors associated with PEMAT Understandability scores among short videos related to precancerous cervical lesions.

| Variables | Multivariable analyses | |
| --- | --- | --- |
|  | β (95% CI) | *p*-value |
| Platforms |  | <0.001 |
| TikTok | Reference |  |
| Bilibili | -0.29 (-0.39, -0.23) |  |
| Kwai | -0.09 (-0.19, -0.07) |  |
| Xiaohongshu | -0.09 (-0.13, -0.05) |  |
| Publication years (per year increase) | 0.012 (0.004, 0.023) | 0.011 |
| Uploader types |  | 0.008 |
| Gynecologists | Reference |  |
| Non-gynecologists | -0.04 (-0.11, 0.03) |  |
| Traditional Chinese medical experts | -0.10 (-0.17, -0.04) |  |
| Science communicators | -0.09 (-0.16, -0.03) |  |
| Patients and family members | -0.15 (-0.23, -0.04) |  |
| Video duration (per minute increase) | 0.011 (-0.002,0.028) | 0.098 |
| Video content |  | 0.031 |
| Basic knowledge about diseases | Reference |  |
| Prevention | -0.07 (-0.16, -0.01) |  |
| Treatment | -0.03 (-0.09, 0.00) |  |
| Posttreatment caveats and reexamination | -0.05 (-0.17, 0.02) |  |
| Others | -0.18 (-0.23, -0.04) |  |
| Video presentation form |  | 0.028 |
| Expert monologs | Reference |  |
| Dialogues | -0.04 (-0.08, -0.01) |  |
| Video blogs of patients | -0.08 (-0.16, -0.04) |  |
| Visual pictures and literature | -0.06 (-0.13, -0.02) |  |
| Surgical operation demonstration | -0.05 (-0.09, -0.03) |  |
| Others | -0.15 (-0.27, -0.08) |  |

**Supplementary Table 8.** Multivariable analyses of factors associated with PEMAT Actionability scores among short videos related to precancerous cervical lesions.

| Variables | Multivariable analyses | |
| --- | --- | --- |
|  | β (95% CI) | *p*-value |
| Platforms |  | <0.001 |
| TikTok | Reference |  |
| Bilibili | -0.33 (-0.38, -0.27) |  |
| Kwai | -0.03 (-0.08, -0.01) |  |
| Xiaohongshu | -0.29 (-0.33, -0.11) |  |
| Publication years (per year increase) | 0.007 (0.001, 0.013) | <0.001 |
| Uploader types |  | 0.019 |
| Gynecologists | Reference |  |
| Non-gynecologists | -0.06 (-0.13, -0.03) |  |
| Traditional Chinese medical experts | -0.12 (-0.27, -0.05) |  |
| Science communicators | -0.14 (-0.22, -0.06) |  |
| Patients and family members | -0.15 (-0.28, -0.03) |  |
| Video duration (per minute increase) | 0.014 (-0.002, 0.030) | 0.084 |
| Video content |  | 0.041 |
| Basic knowledge about diseases | Reference |  |
| Prevention | -0.03 (-0.21, -0.02) |  |
| Treatment | -0.08 (-0.12, -0.03) |  |
| Posttreatment caveats and reexamination | -0.05 (-0.18, -0.01) |  |
| Others | -0.14 (-0.23, -0.05) |  |
| Video presentation form |  | 0.066 |
| Expert monologs | Reference |  |
| Dialogues | -0.05 (-0.10, 0.06) |  |
| Video blogs of patients | -0.12 (-0.23, 0.02) |  |
| Visual pictures and literature | -0.09 (-0.18, -0.01) |  |
| Surgical operation demonstration | -0.04 (-0.17, -0.03) |  |
| Others | -0.14 (-0.22, -0.01) |  |

**Supplementary Table 9.** Multivariable analyses of factors associated with higher HONCODE scores among short videos related to precancerous cervical lesions

| Variables | Multivariable analyses | |
| --- | --- | --- |
|  | OR (95% CI) | *p*-value |
| Platforms |  | <0.001 |
| TikTok | Reference |  |
| Bilibili | 0.34 (0.24, 0.42) |  |
| Kwai | 0.61 (0.46, 0.91) |  |
| Xiaohongshu | 0.39 (0.29, 0.53) |  |
| Publication years (per year increase) | 1.13 (1.06, 1.22) | 0.025 |
| Uploader types |  | 0.006 |
| Gynecologists | Reference |  |
| Non-gynecologists | 0.83 (0.57, 0.95) |  |
| Traditional Chinese medical experts | 0.46 (0.31, 0.72) |  |
| Science communicators | 0.62 (0.39, 0.97) |  |
| Patients and family members | 0.50 (0.32, 1.09) |  |
| Video duration (per minute increase) | - | - |
| Video content |  | 0.020 |
| Basic knowledge about diseases | Reference |  |
| Prevention | 0.71 (0.42, 1.17) |  |
| Treatment | 0.88 (0.63, 0.96) |  |
| Posttreatment caveats and reexamination | 0.54 (0.51, 0.99) |  |
| Others | 0.35 (0.32, 0.91) |  |
| Video presentation form |  | <0.001 |
| Expert monologs | Reference |  |
| Dialogues | 0.71 (0.55, 1.92) |  |
| Video blogs of patients | 0.56 (0.33, 0.71) |  |
| Visual pictures and literature | 0.78 (0.46, 0.99) |  |
| Surgical operation demonstration | 0.65 (0.39, 0.85) |  |
| Others | 0.40 (0.18, 0.83) |  |

**Supplementary Table 10.** Audience engagement metrics stratified by uploader type, content category, and presentation format.

| Variables | Likes | Comments | Collections | Shares |
| --- | --- | --- | --- | --- |
| Medical practitioners, median (IQR) |  |  |  |  |
| Yes | 115 (9, 467) | 11 (1, 69) | 22 (3, 90) | 20 (2, 89) |
| No | 66 (8, 562) | 8 (0, 175) | 25 (4, 249) | 17 (2, 93) |
| Uploader types, median (IQR) |  |  |  |  |
| Gynecologists | 127 (11, 493) | 12 (1, 73) | 24 (3, 94) | 21 (3, 92) |
| Non-gynecologists | 3 (0, 18) | 0 (0, 2) | 1.00 (0, 4) | 0 (0, 6) |
| Traditional Chinese medical experts | 18 (2, 61) | 2 (0, 16) | 3 (1, 13) | 3 (0, 9) |
| Science communicators | 12 (5, 35) | 1 (0, 3) | 6 (3, 29) | 4 (1, 20) |
| Patients and family members | 587 (157, 1214) | 198 (93, 453) | 244 (38, 582) | 78 (16, 279) |
| Video content, median (IQR) |  |  |  |  |
| Basic knowledge about diseases | 291 (70, 811) | 34 (5, 142) | 54 (1, 175) | 52 (12, 182) |
| Prevention | 10 (2, 180) | 1 (0, 9) | 3 (1, 35) | 2 (0, 26) |
| Treatment | 54 (5, 357) | 7 (0, 48) | 14 (2, 63) | 11 (1, 66) |
| Posttreatment caveats and reexamination | 86 (12, 291) | 20 (3, 81) | 14 (2, 47) | 11 (2, 37) |
| Others | 19 (6, 51) | 3 (0, 9) | 8 (3, 30) | 6 (1, 21) |
| Video presentation form, median (IQR) |  |  |  |  |
| Expert monologs | 157 (14, 518) | 14 (1, 71) | 29 (5, 98) | 28 (3, 104) |
| Dialogues | 34 (3, 228) | 5 (0, 47) | 8 (1, 50) | 5 (0, 46) |
| Video blogs of patients | 605 (169, 1251) | 202 (84, 453) | 169 (44, 509) | 96 (21, 268) |
| Visual pictures and literature | 9 (3, 54) | 0 (0, 4) | 6 (2, 34) | 4 (1, 14) |
| Surgical operation demonstration | 508 (69, 4491) | 59 (3, 440) | 63 (8, 673) | 55 (4, 603) |
| Others | 13 (6, 71) | 2 (1, 7) | 11 (4, 24) | 6 (2, 22) |

**Supplementary Figure 1.** Global Quality Score (GQS) stratified by uploader characteristics, video content, and presentation form. (A) Comparison of GQS between videos uploaded by medical practitioners and non-medical practitioners; (B) Comparison of GQS among different uploader types; (C) Comparison of GQS across different video content categories; (D) Comparison of GQS across different video presentation forms. Data are presented as median (IQR). Statistical comparisons were performed using the Mann-Whitney U test for two-group comparisons and the Kruskal-Wallis test followed by Dunn’s multiple comparisons test for comparisons among multiple groups.

**Supplementary Figure 2.** JAMA benchmark scores stratified by uploader characteristics, video content, and presentation form. (A) Comparison of JAMA scores between videos uploaded by medical practitioners and non-medical practitioners; (B) Comparison of JAMA scores among different uploader types; (C) Comparison of JAMA scores across different video content categories; (D) Comparison of JAMA scores across different video presentation forms. Data are presented as median (IQR). Statistical comparisons were performed using the Mann-Whitney U test for two-group comparisons and the Kruskal-Wallis test followed by Dunn’s multiple comparisons test for comparisons among multiple groups.

**Supplementary Figure 3.** Modified DISCERN scores stratified by uploader characteristics, video content, and presentation form. (A) Comparison of modified DISCERN scores between videos uploaded by medical practitioners and non-medical practitioners; (B) Comparison of modified DISCERN scores among different uploader types; (C) Comparison of modified DISCERN scores across different video content categories; (D) Comparison of modified DISCERN scores across different video presentation forms. Data are presented as median (IQR). Statistical comparisons were performed using the Mann-Whitney U test for two-group comparisons and the Kruskal-Wallis test followed by Dunn’s multiple comparisons test for comparisons among multiple groups.

**Supplementary Figure 4.** PEMAT understandability scores stratified by uploader characteristics, video content, and presentation form. (A) Comparison of PEMAT understandability scores between videos uploaded by medical practitioners and non-medical practitioners; (B) Comparison of PEMAT understandability scores among different uploader types; (C) Comparison of PEMAT understandability scores across different video content categories; (D) Comparison of PEMAT understandability scores across different video presentation forms. Data are presented as median (IQR). Statistical comparisons were performed using the Mann-Whitney U test for two-group comparisons and the Kruskal-Wallis test followed by Dunn’s multiple comparisons test for comparisons among multiple groups.

**Supplementary Figure 5.** PEMAT actionability scores stratified by uploader characteristics, video content, and presentation form. (A) Comparison of PEMAT actionability scores between videos uploaded by medical practitioners and non-medical practitioners; (B) Comparison of PEMAT actionability scores across different uploader types; (C) Comparison of PEMAT actionability scores among different video content categories; (D) Comparison of PEMAT actionability scores across different video presentation forms. Data are presented as median (IQR). Statistical comparisons were performed using the Mann-Whitney U test for two-group comparisons and the Kruskal-Wallis test followed by Dunn’s multiple comparisons test for comparisons among multiple groups.

**Supplementary Figure 6.** HONcode scores stratified by uploader characteristics, video content, and presentation form. (A) Comparison of HONcode scores between videos uploaded by medical practitioners and non-medical practitioners; (B) Comparison of HONcode scores among different uploader types; (C) Comparison of HONcode scores across different video content categories; (D) Comparison of HONcode scores across different video presentation forms. Data are presented as median (IQR). Statistical comparisons were performed using the Mann-Whitney U test for two-group comparisons and the Kruskal-Wallis test followed by Dunn’s multiple comparisons test for comparisons among multiple groups.
